# The Role of Heterocysts in Cyanotoxin Production during Nitrogen Limitation

**DOI:** 10.3390/toxins15100611

**Published:** 2023-10-13

**Authors:** Mohamed N. Gomaa, Wayne W. Carmichael

**Affiliations:** 1 Department of Biochemistry, College of Science, University of Jeddah, Jeddah 21589, Saudi Arabia; 2 Department Biological Sciences (Emeritus), Wright State University, Dayton, OH 45435, USA

**Keywords:** harmful algal blooms, nutrient management, *Aphanizomenon* sp., saxitoxin, neosaxitoxin

## Abstract

Cyanobacteria harmful algal blooms (cyanoHABs) can have impacts on human health, aquatic ecosystems, and the economy. Nutrient management is an important mitigation and even remediation strategy. In this work, the paralytic shellfish toxin (PST)-producing *Aphanizomenon* (*Aphan*.) *flos-aquae* (Linnaeus) Ralfs ex Bornet & Flahault (now identified as *Aphan.* sp.) single filament isolate NH-5 was grown in P-depleted media, N-depleted media, and complete BG-11 media. Growth and heterocyst and vegetative cells were monitored using dry weight and cell counts. Ultrasonication was used to separate heterocysts from vegetative cells. HPLC-FLD with post-column derivatization was used to determine the saxitoxin (STX) and neosaxitoxin (NEOSTX) concentration per cell. *Aphan.* sp. NH-5 biomass was lower in the P-depleted media than in the N-depleted media and the control, though higher heterocyst counts were detected in the N-depleted media. The heterocyst toxin concentration was significantly higher compared to the vegetative cells for the N-depleted media, control, and P-depleted media. However, no significant differences were found among all preparations with regard to the STX-to-NEOSTX ratio. We conclude that N limitation induced higher heterocyst numbers and that N fixation activity is a factor behind the increase in the STX and NEOSTX production of *Aphan.* sp. NH-5.

## 1. Introduction

A general definition of harmful algal blooms is given by Watson and Molet [1] “An Algal bloom is a rapid increase or accumulation of free-floating and attached eukaryotic algae or prokaryotic cyanobacteria (formerly known as blue-green algae) commonly but not usually in a surface waterbody, frequently produced by one or a few species”. CyanoHAB is a specific term for harmful cyanobacteria that cause problems in aquatic ecosystems. Cyanobacteria are prokaryotic phytoplankton formerly called blue–green algae. Their problems are increased when cyanoHABs produce toxins [2]. 

Phosphorous (P) is an important limiting factor for primary production in plants including prokaryotic cyanobacteria [3]. Nitrogen (N) and nitrogen limitation are also important in regulating how N-fixing cyanobacteria function [4].

*Aphanizomenon* (*Aphan.*), a filamentous, potential toxin-producing cyanobacterium, is differentiated into vegetative cells, akinetes (resting spores), and heterocyst cells responsible for N fixation [5]. Cyanobacteria form heterocyst cells to counteract N deficiency and maintain anaerobic conditions appropriate for N fixation through morphological and physiological restructuring [6]. Heterocysts are cells within filaments that differentiate into large, round to oblong cells that fix N using the oxygen-sensitive enzyme nitrogenase during dark periods of light–dark growth cycles under anaerobic conditions [7]. Vegetative and heterocyst cells share metabolic efforts by exchanging sugars, and N heterocysts are often found in central areas of the filament, a typical position for *Aphanizomenon*. This characteristic, in combination with other features including cells that consolidate into linear (non-branching) chains called trichomes and parallel trichomes that unite into aggregates called rafts, is used to identify this genus [8]. The ratio of heterocysts to vegetative cells usually ranges from 1:10 to 1:20 [9]. The number of heterocysts increases with decreasing available nitrate-nitrogen when *Aphanizomenon* is grown in ASM-1 medium [10]. 

The first report of a toxin-producing strain of *Aphan.* sp. was in 1980 [11], with the isolation and culture of strain NH-5 producing neosaxitoxin (NEOSTX) (93%) and saxitoxin (STX) (7%) [12,13,14]. To date, the only neurotoxins produced by the NH-5 strain are STX and NEOSTX [15]. 

A positive relationship between growth and nitrogen availability was reported during the growth of *Protogonyaulax* (a non-N fixer eukaryotic dinoflagellate) [16]. However, the toxin production per cell was about the same in all conditions. 

Lower levels of nitrate-nitrogen cause a decrease in growth and lower microcystin production of *Microcystis aeruginosa* (a non-N-fixing prokaryotic cyanobacteria) and *Oscillatoria agardhii* (a N-fixing cyanobacterium that does not have heterocysts). Conversely, increased levels of nitrate-nitrogen cause an increase in growth and microcystin production in both of these genera [17,18,19,20,21]. 

An inverse relationship between available nitrate-N in the culture media and the amount of toxin per cell was first demonstrated [15] when using N-depleted BG-11 media to grow *Aphan.* sp. (N-fixing heterocyst-forming prokaryotic cyanobacteria). This thesis work [15] also reported no significant difference in biomass production between N-depleted and non-depleted cultures. The work suggested that the increase in toxin content per mg cells in the N-depleted media may be due to an increased number of heterocysts. Such an inverse relationship between the nitrate availability in synthetic media and the toxin concentration per cell was confirmed during the culturing of *Anabaena flos-aquae* and *Aphan. flos-aquae*, capable of producing the neurotoxin Anatoxin-a [22]. This was confirmed by a study showing that N starvation could increase Anatoxin production [23]. 

This inverse relationship was also demonstrated when cultures of *Cylindrospermopsis raciborskii* (a N-fixing heterocyst-forming cyanobacterium) cells were grown in the absence of N, with cylindrospermopsin being increased [24]. Similarly, N limitation caused an increase in the nodularin concentration produced by *Nodularia spumigena* GR8b (a N-fixing heterocyst-forming cyanobacterium) [25]. 

This current work was designed to test our hypothesis that the reason for the inverse relationship between N availability and increasing toxin production, in the heterocyst-forming NH-5, might be due to increasing toxin content within the heterocyst compared with those of the vegetative cells.

## 2. Results

### 2.1. Growth Curve Profile during Nitrogen (N) and Phosphorus (P) Starvation

**Figure 1:**
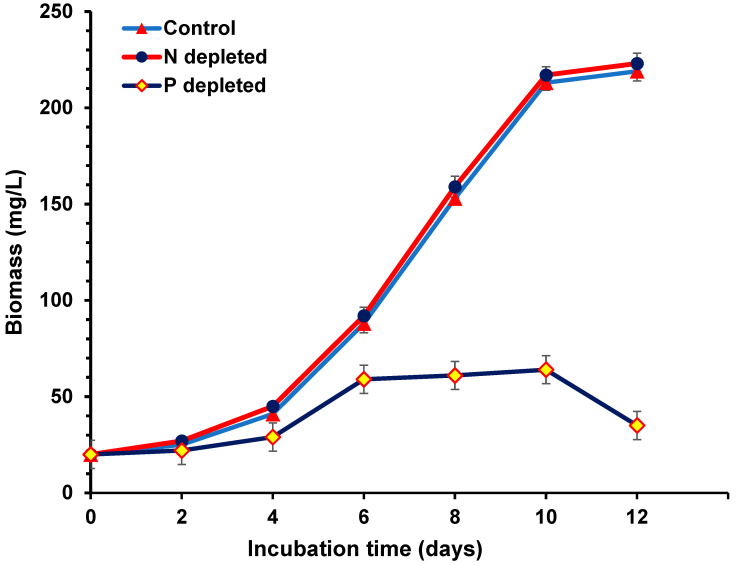
Growth profile of *Aphan.* sp. *NH-5* during nitrogen and phosphorus starvation.

The growth curve profile of nitrogen-depleted, P-depleted, and non-depleted (control) cultures during the culturing period of 12 days indicated no significant differences between the depleted nitrogen and control cultures (Figure 1). However, a significantly lower profile was observed in the P-depleted cultures (Figure 1). The stationary phase of the growth curve was reached after 10 days of incubation of the depleted nitrogen and control cultures. The experiment was concluded on the 12th day before detecting the decline phase. The stationary phase of the growth curve was reached after 6 days of incubation of the P-depleted cultures, and the decline phase was detected after 10 days of incubation. 

### 2.2. Heterocyst/Vegetative Ratio during N and P Starvation

**Figure 2:**
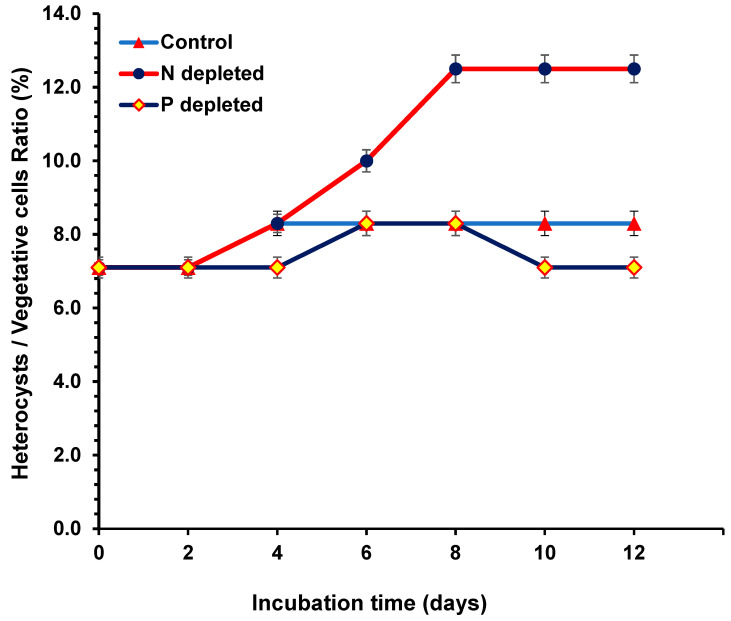
Heterocyst/vegetative ratio of *Aphan.* sp. *NH-5* during nitrogen and phosphorus starvation compared with the control cultures.

After the cell counts for the different cultures, the ratio of heterocyst to vegetative cells observed in this study ranged from 1:12 to 1:8. Maximum ratios were observed during the log phase of all treatments and dropped to 1:14 during the stationary phase. Significant differences were detected in the heterocyst/vegetative cell ratio for the 0% N cultures compared with the 0% P and control cultures (Figure 2). 

### 2.3. Toxin Concentrations during Nitrogen and Phosphorus Starvation

**Figure 3:**
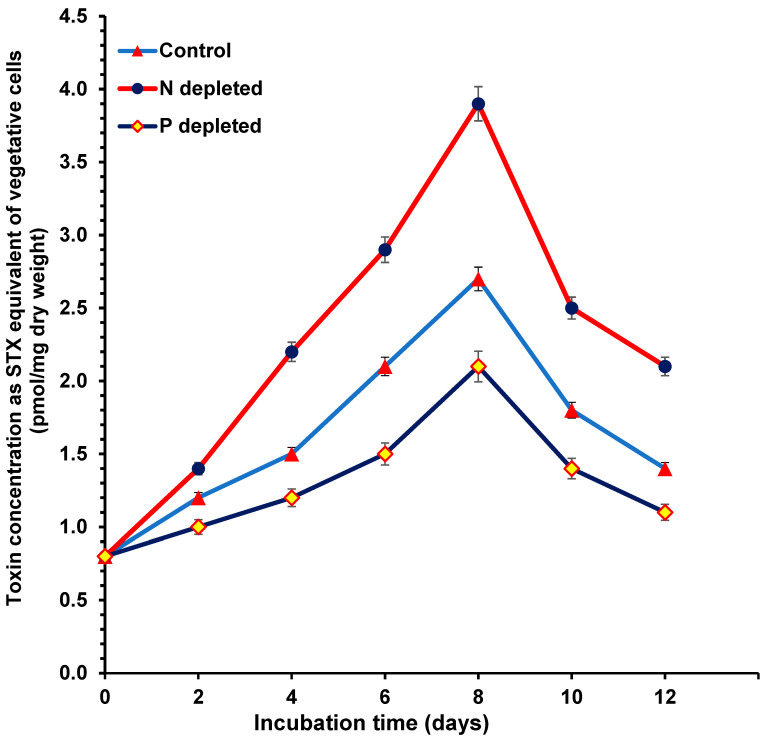
Toxin concentration as STX equivalent of *Aphan.* sp. vegetative cells during the incubation period of nitrogen- and phosphorus-depleted cultures compared with the control cultures.

**Figure 4:**
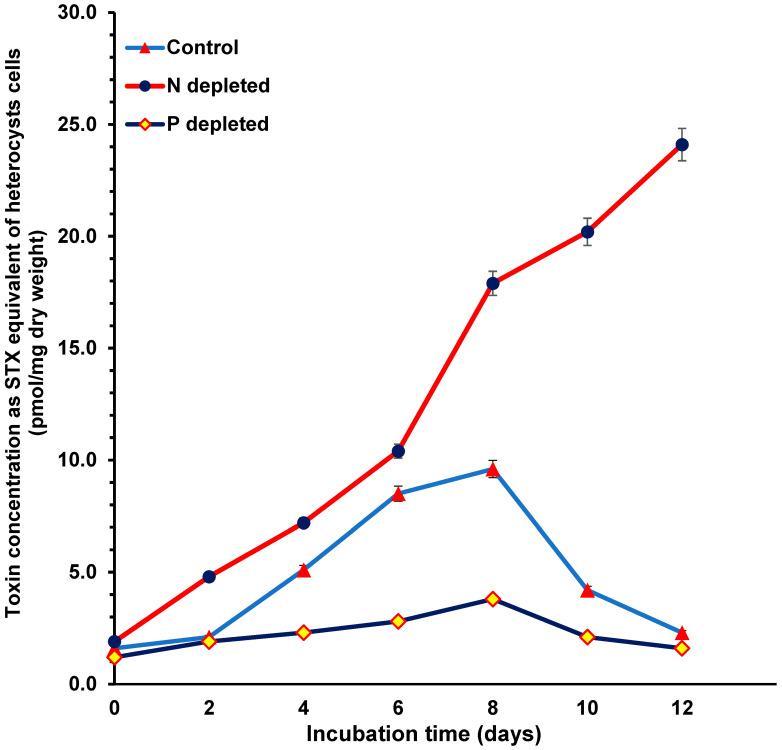
Toxin concentration as STX equivalent of *Aphan.* sp. heterocyst cells during the incubation period for nitrogen- and phosphorus-depleted cultures compared with the control cultures.

The toxin concentrations, calculated as STX equivalent in pmol/mg dry weight of the vegetative cells (Figure 3) and heterocyst cells (Figure 4), of all treatments were determined using HPLC-FLD with post-column derivatization. The STX equivalent concentrations of both the vegetative cells (Figure 3) and heterocyst cells (Figure 4) were higher in the 0% N cultures followed by the control cultures, whereby the 0% P cultures contained the lowest toxin levels. The STX equivalent content of the heterocyst cells was five times higher than the vegetative cells in all treatments.

The STX equivalent concentrations for the different treatments and cell types were similar except for the heterocyst cells in 0% N. The maximum STX equivalent concentration of all treatments occurred after 8 days of incubation (during the end of the log phase of the growth curve) and then declined sharply except for the heterocyst cells at 0% N, which continued in an increasing pattern (no decline was detected before the end of the experiment). For all treatments during the sampling points, the STX equivalent content in the heterocyst cells was higher than in the vegetative cells. The maximum difference was found after 8 days for the vegetative cells (Figure 3) and after 12 days for the heterocysts (Figure 4).

### 2.4. Toxin Ratio Profile during Nitrogen and Phosphorus Starvation

HPLC-FLD with post-column derivatization determination was also used to calculate the ratio of STX/NEOSTX in all treatments. No differences were found between the STX/NEOSTX ratio of the vegetative cells and the heterocyst cells in all treatments. This means that when the STX concentration is increased, NEOSTX increases and vice versa. The ratio was not affected by the type of cell or the level of nitrogen in the culture. 

## 3. Discussion

As expected, the availability of phosphate is required for the growth of *Aphan.* sp. as shown in this current study and confirmed by de Figueiredo et al. [26], who reported a cyanobacterial bloom of *Aphan.* sp. occurrence in the presence of sufficient P and nitrogen limitation in the water throughout their study period, while under P depletion, the *Aphan.* sp. occurred in low densities. This suggests that this strain is not able to dominate in phosphate-depleted conditions. This P dependence has also been reported for this genus by others [27,28].

However, the results of our current study showed that N limitation did not affect the growth of *Aphan.* sp. as previously reported during an *Aphan.* sp. bloom in a N-limited bloom, which means that this species is not N dependent, due to its capability of N-fixing [29]. Our results support these reports, of lower biomass in P-depleted cultures, but there were no significant differences for the N-depleted cultures compared with the complete cultures.

The increase in PST production per mg cells reported in this study under N starvation was supported by others [15] working with STXs produced by *Aphan.* sp., [22], anatoxin-a produced by *Anabaena flos-aquae* and *Aphan.* sp. [24] working with cylindrospermopsin, which was produced by *Cylindrospermopsis raciborskii,* and [25,30] working with nodularin produced by *Nodularia spumigena*. All of the organisms mentioned above are nitrogen-fixing heterocyst-forming cyanobacteria.

The decrease in total toxin content as a result of N limitation reported by [16], working with STXs produced by *Protogonyaulax* (a non-N fixer eukaryotic dinoflagellate), was due to lower growth. However, the toxin production per cell was about the same in all conditions. The same positive relationship between N level and microcystin produced by *Microcystis aeruginosa* (a non-nitrogen fixer prokaryotic cyanobacterium) and *Oscillatoria agardhii* (a nitrogen-fixing prokaryotic cyanobacterium that does not have heterocysts) was confirmed in several reports [17,18,19,20,21]. It should be noted that *Microcystis* showed another pattern of response toward nitrogen limitation as the less toxic, high nitrogen Microcystin-RR (MC-RR) dominated under nitrogen-replete conditions, whereas the more toxic, less nitrogen MC-LA dominated under nitrogen-limited conditions [31]. 

However, a third group of organisms with regard to their response to the N level was reported by [32], who observed decreased levels of saxitoxin in response to higher concentrations of nitrogen in the non-N-fixing species *Raphidiopsis brookii*. This report was controversial as it contradicts the two abovementioned groups of organisms (heterocysts and non-heterocyst organisms) and their response to nitrogen limitation.

The function of heterocysts in N fixation is well known, and our results obtained from *Aphan.* sp. *NH-5* in N-depleted BG-11 media align with previous work [33,34], which indicated that the number of heterocysts correlates with the N_2_ fixation activity of the population. Also, a higher number of heterocysts during lower N in ASM-1 medium, used to culture *Aphan*., was observed [9].

From our results and the abovementioned reports, there could be another function for this specialized cell, namely its role in toxin production during N limitation. This role can explain the higher PST production reported in our study from lowering N availability in this heterocyst-forming organism. Also, these reports demonstrate that it is not the type of organism nor the type of toxin that is important, but whether it is a heterocyst or non-heterocyst-forming organism during toxin production in N limitation conditions. 

Our results showed the higher toxin content of the heterocyst (about five times higher) compared to the toxin content of the vegetative cells, which agreed with the explanation by [15], who related the increase in toxin content per mg cells in nitrogen-depleted media to the increase in the number of heterocysts. This means that decreasing the available nitrate in the media would increase the number of heterocysts and, consequently, the amount of toxin per mg cells of the whole culture compared with the non-depleted N culture. The relationship between heterocysts and nitrogen availability by two different strains of *Nodularia spumigena* and *Anabaena* was observed because of the existence of the binding site for the nitrogen-responsive regulatory protein in the heterocyst-forming genes of these organisms, which might explain the function of nitrogen fixation in the synthesis of the toxin [30]. 

Regarding toxin synthesis, it may follow the same mechanism in both vegetative and heterocyst cells. This can explain our results, which showed that the toxin concentrations of both vegetative and heterocyst cells were higher in N-depleted cultures along with the result that showed that the STX/NEOSTX ratios were the same in all treatments. Also, we propose that the toxins might be moved from the heterocyst to the vegetative cells during lower N, which explains the higher toxin concentration in the vegetative cells of the N-depleted cultures. 

In our study, as well as others indicating the effect of N starvation on the production of cyanotoxins under laboratory conditions [23], it is not known how these effects are controlled at a molecular level and how this explains actual responses in the environment. Ecosystem macronutrient eutrophication along with global climate change can also be responsible for the frequency and intensity of cyanotoxic blooms in the ecosystem; however, it seems that for most of the lab experiments explored for cyanobacteria, toxin production is a direct function of the cell propagation rate [23]. The important data gained from laboratory experiments regarding the factors affecting cyanotoxin production are not enough to understand what happens in the field and that is why more field experiments are needed [35]. Because of variations in ecosystems, it is not easy to establish a direct relationship between macronutrient availability and cyanotoxin production. For example, the effect of N limitation might have an important impact on explaining species composition and toxin production differences in the presence of enough P in different ecosystems as lakes become more eutrophic [36]. 

## 4. Conclusions

We, along with others, have observed increasing cyanotoxin production by certain species of cyanobacteria during growth under nitrogen limitation. During the course of our research, we discovered that all the cyanobacterial species that exhibit this are capable of fixing nitrogen using heterocysts. Therefore, we investigated our hypothesis that heterocysts are involved. Our results showed that the heterocysts contained more toxins than the vegetative cells and that more heterocysts were found in the nitrogen-depleted cultures, which explained the increased level of cyanotoxins during nitrogen limitation. We conclude that heterocyst cells were the major factor accounting for greater STX and NEOSTX production during N starvation in the strain NH-5. We postulate that the greater toxin concentration in the vegetative cells could be due to the exchange of toxins during N limitation. The same mechanism for toxin synthesis is assumed to occur in both types of cells as the same toxin ratio profile was detected in N-depleted and non-depleted cultures at all data points. It is important to follow up this work with research at the molecular level. We think that our discovery can help explain variable cyanotoxin production and even assist in the mitigation of cyanoHABs.

## 5. Materials and Methods

### 5.1. Cultures of Aphan sp. NH-5

The organism under study is *Aphanizomenon* (*Aphan.*) *flos-aquae* NH-5 [37], now identified as *Aphan.* sp. [38]. This revised taxonomy of *Aphan. flos-aquae* strain NH-5 was studied by comparing this strain with six other strains of Aphanizomenon. Both the 16S rRNA gene sequences and morphological features were used for comparison. The lower similarities (less than 97.5%) of strain NH-5 in the 16S rRNA gene sequences compared to the *Aphan. flos-aquae* strains and the association within a phylogenetic tree constructed from 16S rRNA gene sequences along with the absence of bundle formation in the trichomes, and the position of akinetes following heterocytes confirm that strain NH-5 was improperly identified as *Aphan. flos-aquae* [38]. Also, the taxonomic status of the ADA clade, Nostocales, was discussed in a recent report [39], whereby a comparative genomics investigation of the ADA clade showed that strain and species differentiation is accompanied by substantial differences in gene content.

BG-11 media [40,41] were used to culture *Aphan* sp. strain NH-5. The media component (Fisher Scientific Chemical, Pittsburgh, PA, USA) is listed in Table 1 and Table 2. BG-11 medium contains a relatively high concentration of nitrate and phosphate. 

Three sets of media were prepared; one was used as a control with complete nitrate (1.5 g/L) and complete phosphate (0.04 g/L). The other two sets of media were prepared, one without nitrate and the other without phosphate. Continuous illumination from cool white, fluorescent lighting (Philips, Dayton, OH, USA) 90 µE·m^−2^·s^−1^, photosynthetically active irradiation, and 20 °C were used to incubate the cultures of *Aphan.* sp. for 12 days.

#### 5.1.1. Preparations of Nitrogen (N)- and Phosphorus (P)-Depleted Cultures

N and P depletion in the cells of *Aphan.* sp. was not achieved from the first batch of cultures prepared without N and P as cyanobacteria store these two elements within their cells. Therefore, two sets of cultures were grown for two weeks, one in 0% N, 100% P (0.04 g/L) media and the other set in 0% P, 100% N (1.5 g/L) media. These cultures were used to inoculate a second batch of 0% N, 100% P media, and 0% P, 100% N media. The second batch of cultures consisted of three replicates of 1.5 L media in a 2 L vessel for each treatment and were grown for 12 days.

#### 5.1.2. Determination of Dry Weight and Cell Count

To determine the dry weight as well as the counts of heterocysts and vegetative cells, three samples with a volume of 10 mL were collected from each treatment every other day. The number of heterocysts and vegetative cells was counted using an Improved Neubauer Haemocytometer (0.1 mm deep) counting chamber [42] under a binocular phase contrast light microscope (Nikon series Optiphot Microscope 150, Nikon Instruments Inc., Melville, NY, USA). The samples were then filtered through a previously weighed Gelman 0.45 µm membrane filter for dry weight determination using a Millipore filtration system (Merck Millipore Life science brand, Burlington, MA, USA) and a suitable vacuum pump. The membrane filters were dried in an oven at 60 °C overnight, reweighed, and the difference in weight was recorded. 

### 5.2. Heterocyst Separation

The heterocyst separation method [43] was used to separate the heterocysts from the vegetative cells. A 150 mL sample was collected every other day after inoculation to accomplish separation. Ultra-sonication was used to separate the thicker walled heterocyst cells from the vegetative cells since the heterocysts resisted the sonication procedure. Cells from the 150 mL samples were centrifuged (Fisher microcentrifuge, Fisher Scientific, Pittsburgh, PA, USA) at 4000× *g* for 5 min and sonicated at level 3.5 for 20 s using a Heat System Ultrasonic sonicator (model W 200 R, Ultrasonics, Inc., Ferndale, WA, USA). This lysed the vegetative cells and left the heterocysts intact. Microscopy was used to confirm the complete lysing of the vegetative cells and the remaining intact cells of the heterocysts. Centrifugation at 4000× *g* for 15 min was used to separate the intact heterocyst cells as a pellet from the supernatant containing the lysed vegetative cells. To lyse the heterocyst cells, the pellet was re-suspended in 2 mL acetic acid and then freeze–thawed three times followed by ultra-sonication at power level 7 for 1 min. 

### 5.3. Saxitoxin and Neosaxitoxin Determination by HPLC-FLD with Post-Column Derivatization

Vegetative and heterocyst cell preparations were used for toxin determination by Oshima et al.’s HPLC method [44] and for the calculation of the STX/NEO ratio. Yasukatsu Oshima of Tohoku University (Sendai, Japan) kindly provided the STX and NEOSTX standards. High-performance liquid chromatography with post-column fluorescent derivatization (HPLC–FLD) was performed using a Waters HPLC system (Waters Corporation, Milford, MA, USA) equipped with a 600E pump system and two post-column pumps. A guard column with dimensions of 5 mm length, 4.6 mm diameter, and 5 µm particle size was positioned before an Inertsil Reverse Phase C8 column with a dimension of 250 mm length, 4.6 mm diameter, and 5 µm particle size. This was used to separate the STX and NEOSTX. Five µL of STX and NEOSTX standards followed by 5 µL of each sample were injected into the system. The isocratic mobile phase consisted of 30 mM of ammonium phosphate buffer containing 2 mM of sodium 1-heptasulfonate at pH 7.1 mixed with acetonitrile in a ratio of 96/4 (*v*/*v*) used to complete the separation. Post-column derivatization, also known as post-column reaction, was used to visualize the STX and NEOSTX using a fluorescence detector. The post-column reaction system mixed the stream of eluents from the HPLC column with a stream of reagent solution. The mixture flowed through a reactor to allow enough time for the chemical reactions to complete. The slow reaction was sped up by heating. The post column reaction system used was composed of a double plunger pump to mix the oxidizing reagent and ortho-periodic acid as an acidifier with the eluents coming out of the column. This system was connected to a heating coil set at 90 °C. The Waters 470 fluorescence detector was set at an excitation wavelength of 340 nm, a 15 nm slit, and an emission wavelength of 400 nm; a 20 nm slit was used to detect the separated toxins. Waters 2010 Millennium Chromatography Manager was used for data collection and integration.

### 5.4. Quantitation and Calibration of Saxitoxin and Neosaxitoxin Determination by HPLC-FLD with Post-Column Derivatization

For the quantitation analysis of STX and NEOSTX, reference solutions at concentrations ranging from 0.3 to 4 µM were prepared in 0.05 M acetic acid and kept at −40 °C until use. To prepare the calibration curve, 5 µL aliquots of different concentrations of the standards were injected prior to sample injection of 5 µL. A calibration curve was prepared. Injections of only one reference material were repeatedly injected between each five injections of samples to match the retention times and peak areas. Calculation of toxin concentration was carried out using Waters 2010 Millennium Chromatography Manager for data collection and integration. For the effect of nutrients on toxin production study, the total toxin concentration was calculated as STX equivalent. For the calculation of the STX/NEOSTX ratio, peak areas were used to calculate the ratio. 

### 5.5. Statistical Analysis

Differences in dry weight and the heterocyst/filament ratio as well as differences between the toxin concentrations of *Aphan.* sp. NH-5 vegetative and heterocyst cell preparations grown in the 0% nitrate and 100% nitrate media were analyzed by one-way analysis of variance using the general linear model of SAS [45]. Mean separation was carried out using Duncan’s multiple range test [46].

## Figures and Tables

**Table 1:** Trace metal components to be added to the BG-11 media.

No.	Component	Stock Solution	Quantity	Molar Concentration in Final Medium
1.	MgNa_2_ EDTA 3H_2_O	--------	1.000 g	2.26 µM
2.	H_3_BO_3_	--------	2.860 g	46.3 µM
3.	MnCl_2_ 4H_2_O	--------	1.810 g	9.15 µM
4.	ZnSO_4_ 7H_2_O	--------	0.220 g	0.765 µM
5.	CuSO_4_ 5H_2_O	79.0 g L^−1^ H_2_O	1 mL	0.316 µM

All chemicals are from Fisher Scientific Chemical, Pittsburgh, PA, USA.

**Table 2:** BG-11 media constituents and the sequence that should be followed to prepare the medium to avoid precipitation of its constituents.

No.	Constituent	Volume	Concentration of the Stock Solution	Final Molar Concentration
1	NaNO_3_	10 mL/L	30 g/200 mL dH_2_O	17.6 mM
2	K_2_HPO_4_	10 mL/L	0.8 g/200 mL dH_2_O	0.23 mM
3	MgSO_4_ 7H_2_O	10 mL/L	1.5 g/200 mL dH_2_O	0.3 mM
4	CaCl_2_ 2H_2_O	10 mL/L	0.72 g/200 mL dH_2_O	0.24 mM
5	Citric Acid H_2_O	10 mL/L	0.12 g/200 mL dH_2_O	0.031 mM
6	Ferric Ammonium Citrate	10 mL/L	0.12 g/200 mL dH_2_O	0.021 mM
7	Na_2_EDTA 2H_2_O	10 mL/L	0.02 g/200 mL dH_2_O	0.0027 mM
8	Na_2_CO_3_	10 mL/L	0.4 g/200 mL dH_2_O	0.19 mM
9	BG-11 Trace Metals Solution	1 mL/L		

All chemicals are from Fisher Scientific Chemical, Pittsburgh, PA, USA.

## Data Availability

The data presented in this study are available on request from the corresponding author. The data were not made publicly available since it was done before public data bases were set up.
